# Bacteria and cancer: cause, coincidence or cure? A review

**DOI:** 10.1186/1479-5876-4-14

**Published:** 2006-03-28

**Authors:** DL Mager

**Affiliations:** 1 The Forsyth Institute, 140 The Fenway, Boston, MA, USA

## Abstract

Research has found that certain bacteria are associated with human cancers. Their role, however, is still unclear. Convincing evidence links some species to carcinogenesis while others appear promising in the diagnosis, prevention or treatment of cancers. The complex relationship between bacteria and humans is demonstrated by *Helicobacter pylori *and *Salmonella typhi *infections. Research has shown that *H. pylori *can cause gastric cancer or MALT lymphoma in some individuals. In contrast, exposure to *H. pylori *appears to reduce the risk of esophageal cancer in others. *Salmonella typhi *infection has been associated with the development of gallbladder cancer; however *S. typhi *is a promising carrier of therapeutic agents for melanoma, colon and bladder cancers. Thus bacterial species and their roles in particular cancers appear to differ among different individuals. Many species, however, share an important characteristic: highly site-specific colonization. This critical factor may lead to the development of non-invasive diagnostic tests, innovative treatments and cancer vaccines.

## Introduction

An overwhelming body of evidence has determined that relationships among certain bacteria and cancers exist. The bacterial mechanisms involved are as yet unclear. These gaps in knowledge make it impossible to state the exact progression of events by which specific bacteria may cause, colonize or cure cancer. Therefore, many questions remain. For example, why do infections that are wide spread appear to cause cancer in only a minority of individuals? Do certain infective agents initiate or promote cancer or does an early undetected cancer facilitate the acquisition of the infection? Can the exposure to or colonization of specific bacteria prevent or treat certain cancers? Can the highly site specific colonization of certain bacteria for a tumor be clinically useful in the diagnosis of cancer or delivery of a therapeutic agent?

The scope of this review is broad therefore a wide range of reports is presented. Recent findings that have found associations between certain bacterial infections and tumor development will be discussed as well as genetic factors that may predispose individuals to "cancer- causing" infections. Mechanisms thought to be involved with the carcinogenic, diagnostic and preventive or treatment roles of bacteria are introduced. As the carcinogenic potential of viral agents and *H. pylori *has been reviewed extensively elsewhere, it will not be included here.

## Bacteria and carcinogenesis

It is estimated that over 15% of malignancies worldwide can be attributed to infections or about 1.2 million cases per year. Pisani et al. [1] Infections involving viruses, bacteria and schistosomes have been linked to higher risks of malignancy. Although viral infections have been strongly associated with cancers [2,3]  bacterial associations are significant. For example, convincing evidence has linked *Helicobacter pylori *with both gastric cancer and mucosa-associated lymphoid tissue (MALT) lymphoma [4-6], however other species associated with cancers include: *Salmonella typhi *and gallbladder cancer [7-10], *Streptococcus bovis *and colon cancer [11-14] and *Chlamydia pneumoniae *with lung cancer [15-17]. Important mechanisms by which bacterial agents may induce carcinogenesis include chronic infection, immune evasion and immune suppression [18].

It has been shown that several bacteria can cause chronic infections or produce toxins that disturb the cell cycle resulting in altered cell growth [15,16,19]. The resulting damage to DNA is similar to that caused by carcinogenic agents as the genes that are altered control normal cell division and apoptosis [20,21]. Processes that encourage the loss of cellular control may be tumor initiators (directly causing mutations) or promoters (facilitating mutations). Tumorigenesis is initiated when cells are freed from growth restraints, later promotion results when the immune system is evaded favoring further mutations and increased loss of cell control. As the tumor proliferates an increased blood supply is needed resulting in the organization of blood vessels or angiogenesis. Subsequent invasion occurs if the tumor breaks down surrounding tissues. The worst outcome is metastasis which results when cells break away from the tumor and seed tumors at distant sites [8].

The immune system is an important line of defense for tumor formation of malignancies that express unique antigens. Certain bacterial infections may evade the immune system or stimulate immune responses that contribute to carcinogenic changes through the stimulatory and mutagenic effects of cytokines released by inflammatory cells. These include reactive oxygen species (ROS), [22,23], interleukin-8 (IL-8) [11], cyclooxygenase-2 (COX-2), [24], reactive oxygen species (ROS) and nitric oxide (NO) [25]. Chronic stimulation of these substances along with environmental factors such as smoking, or a susceptible host appears to contribute significantly to carcinogenesis.

### *Salmonella typhi *and gallbladder cancer

Worldwide annual incidence of gallbladder cancer (GC) is 17 million cases with high incidence rates in certain populations. The malignancy is usually associated with gallstone disease, late diagnosis, unsatisfactory treatment, and poor prognosis. The five-year survival rate is approximately 32 percent for lesions confined to the gallbladder mucosa and one-year survival rate of 10 percent for more advanced stages [26]. Over 90 percent of gallbladder carcinomas are adenocarcinoma [27] involving gallstones in 78% – 85% of cases [26].

There are several risk factors for gallbladder cancer. The main associated risk factors include cholelithiasis (especially untreated chronic symptomatic gallstones), obesity, reproductive factors, environmental exposure to certain chemicals, congenital developmental abnormalities of the pancreatic bile-duct junction and chronic infections of the gallbladder [26,28]. The interplay of genetic susceptibility, lifestyle factors and infections in gallbladder carcinogenesis is still poorly understood [29], however a link has been specifically proposed between chronic bacterial infections of the gallbladder and *Salmonella typhi *[26].

The strongest epidemiological evidence of bacterial oncogenic potential, aside of *Helicobacter pylori*, concerns *S. typhi*. Infection with this bacterium of typhoid, can lead to chronic bacterial carriage in the gallbladder [30]. Recent epidemiological studies have shown that those who become carriers of *S. typhi *have 8.47 times the increased risk of developing carcinoma of the gallbladder compared with people who have had acute typhoid and have cleared the infection [26]. These findings agreed with earlier investigations by Welton et al. [31] and Caygill [30].

A case-control study by Welton et al [31] compared those who experienced acute infection with *S. typhi *to those who subsequently became chronic carriers following the 1922 typhoid outbreak in New York. Carriers were six times more likely to die of hepatobiliary carcinoma than matched controls. Additional evidence was found in an analysis of the 1964 typhoid outbreak in Aberdeen [30]. Their findings also suggested a strong association between chronic carrier status and hepatobiliary carcinoma. These studies also agreed, people who contracted typhoid but did not become carriers were not at higher risk of cancer [8,26,30,31].

The highest incidence of gallbladder cancer (GC) in the world is among populations of the Andean area, North American Indians, and Mexican Americans. In Europe, the highest rates are found in Poland, the Czech Republic and Slovakia. The high rates observed in Latin America are primarily in populations with high levels of Indian mixture [32]. This evidence supports the notion that increased susceptibility to gallbladder cancer depends on genetic factors that predispose people to gallbladder cancer either as primary factors, or secondarily as promoters by favoring the development of cholesterol gallstones. The highest mortality rates are in South America, (3.5–15.5 per 100,000) and among Mexican Americans [26]. Incidence rates of GC in various ethnic groups in the USA confirmed the worldwide pattern, as GC was substantially more frequent among Hispanic than non-Hispanic white women and men. Interestingly, compared to non-Hispanic whites an excess of GC was also reported among American Indians in New Mexico, in agreement with the excess in incidence rates reported for American Indians and Alaskan Natives [33]. The malignancy is 3 times higher, however, among women than men in all populations [26].

Two main pathways to GC exist worldwide. The predominant pathway involves gallstones and resultant cholecystitis and affects women to a greater extent than men. The risk of developing gallstones in response to environmental factors is genetically determined, as shown by the marked tendency of gallstones to cluster in families [34]. The other pathway involves an anomalous pancreatobiliary duct junction (APBDJ), a congenital malformation of the biliary tract that is more frequent in Japan, Korea, and possibly China, than in Western countries [28]. In APBDJ, the premature junction of common bile and pancreatic ducts results in regurgitation of pancreatic juice into the gallbladder, leading to bile stasis and inflammation, though generally less severe than that resulting from gallstones [28].

Currently the prevention of gallbladder cancer in high risk populations depends upon the diagnosis of gallstones and removal of the gallbladder. Indeed, a strong inverse association between number of cholecystectomies and GC incidence and mortality rates can be found in many countries. The increase of GC mortality reported in Chile in the 1980s was related to decreased rates of cholecystectomies [35]. Increased rates led to the removal of gallbladders at risk, and a reduction of GC incidence and mortality in Europe and the United States [36].

Unfortunately, information about the genetic changes involved in gallbladder carcinogenesis is limited. Most studies have focused on gene abnormalities and deletions ("loss of heterozygosity") at chromosomal regions harboring known or putative tumor suppressor genes [28]. It appears, however, that *TP53 *inactivation has an important and early role in gallbladder carcinoma associated with gallstones and chronic inflammation. This inactivation would abrogate the tumor suppressor function of the p53 protein resulting in impairments in cell cycle control, cellular repair and apoptosis.

In contrast, *KRAS *mutations are frequent and early events in tumors associated with APBDJ [28] but detected less often in gallbladder carcinomas associated with gallstones. KRAS is an oncogene that encodes a protein that is a member of the small GTPase family. A mutation in this gene results in an abnormal protein implicated in several malignancies, including lung adenocarcinoma, ductal carcinoma of the pancreas and colorectal carcinoma among others.

### *Chlamydophila pneumoniae *and lung cancer

Lung cancer is the leading cause of cancer death in the United States and many countries in the Western world. In 2002, the most recent year for which statistics are available, 90,121 males and 67,509 females died from lung cancer [37]. About 6 out of 10 people with lung cancer die within 1 year of finding out they have lung cancer. Between 7 and 8 will die within 2 years [38]. Although patients may experience a partial or complete response to treatment, most patients relapse and die. Increased dosage of chemotherapy or length of treatment has not been beneficial [39].

*Chlamydophila *(formerly *Chlamydia) pneumoniae *infection has been implicated in several chronic lung diseases by serology and direct antigen detection. Acute lower respiratory tract infection caused by *C. pneumoniae *seems often to precede attacks of asthma in both children and adults but is also involved in some exacerbations of chronic bronchitis. More importantly it seems to be strongly associated with chronic obstructive lung disease irrespective of exacerbation status. Moreover, persistently elevated *C. pneumoniae *antibody titers have been observed in sarcoidosis and lung cancer [40].

*C. pneumoniae *is a Gram-negative bacillus and an intracellular parasite that causes respiratory infection in more than 50% of adults. The route of transmission is usually by aerosol and in most cases these infections are mild. The bacterium is, however, an important cause of pneumonia, bronchitis, sinusitis, rhinitis and chronic obstructive pulmonary disease [41]. Respiratory infections from *C. pneumoniae *vary in different countries and populations, being endemic in the United States and epidemic in Scandinavian countries [19].

After acute infection the *C. pneumoniae *intracellular life cycle is characterized by the development of metabolically inert (and thus antibiotic resistant) atypical "persistent" inclusions. These inclusions contain increased quantities of chlamydial heat shock protein 60, a highly immunogenic protein implicated in the pathogenesis of chronic chlamydial infections. The resulting clinical course is acute symptomatic illness followed by chronic respiratory symptoms.  Research also suggests that persistent *C. pneumoniae *inflammation correlates with increased risk of lung cancer [16,17,19]. Prospective and retrospective studies both report that individuals with elevated IgA antibody titers to this organism have 50% to 100% increased lung cancer risk [15].

In a study by Kocazeybek et al. [19] the relationship between chronic *C. pneumoniae *infection and lung carcinoma was examined. A total of 123 patients who were smokers and diagnosed with lung carcinoma based on clinical and laboratory (radiological, cytological) findings were examined. 101 (82.1%) of the cases were male. 70 had small-cell, 28 squamous-cell and 7 large-cell carcinomas, while 18 had adenocarcinoma. 123 healthy controls were matched to the cancer patients by age, gender, duration of smoking and locality.

Blood samples (5 ml) were withdrawn at the time of diagnosis (or enrollment for controls) and 1 month later. Values between IgG ≥512 and IgA ≥40 were set as the criteria for chronic *C. pneumoniae *infections. In male patients with lung carcinoma, IgG antibody titers of ≥512 and IgA antibody titers of ≥40 were found at a higher rate than in the control group, however, this ratio was not significant for female patients. These elevations in antibody titers were found in a total of 62 (50.4 %) cases, 54% of the male patients and 36% of the female patients. Chronic *C. pneumoniae *infections were seen statistically more often in male patients with carcinoma who were aged 55 years or younger than in controls (P < 0.001). No difference was reported between male patients with lung carcinoma over age 55 and controls or in blood titers between female patients and controls.

The relationship between *C. pneumoniae *infection and lung carcinoma was studied by Littman et al. [42] in a large prospective case-control study to investigate whether IgA antibody titers to *C. pneumoniae *were associated with lung cancer risk. A total of 508 pairs were enrolled and included both current and former smokers. Serum was collected at baseline and annually thereafter. Antibody determinations of each lung cancer subject and matched control were tested simultaneously in the same titration series in a blinded fashion. *C. pneumoniae *titers (IgA or IgG) ≥16 were considered seropositive, which was consistent with the cutoff used in other studies. Subjects were matched by age, gender, and smoking status at baseline. The median age of cases and controls was 59 years and about half were women. All subjects were also examined for demographic, lifestyle, dietary, and racial and ethnic factors. Lung cancer subjects had a heavier smoking history than controls.

After adjusting for a history of chronic bronchitis or emphysema, lung cancer subjects were more likely to have IgA titers ≥16 (55.4% vs. 51.3%) and ≥256 (5.1% vs. 2.5%) to *C. pneumoniae *than controls. Individuals with antibody tiers IgA ≥16 had 1.2 times the risk of lung cancer (95% confidence interval, 0.9–1.6) compared to those with lower titers. Investigators reported a significant trend (P = 0.007) of increasing odds ratios with increasing IgA titers primarily due to an odds ratio of 2.8 (95% confidence interval, 1.1–6.7) associated with titers ≥256. Elevated IgA was reported with squamous cell carcinomas and to a lesser extent, for small cell carcinomas and adenocarcinomas. There was no evidence of a stronger association with elevated IgG titers however. Subjects with race not classified as White or Black were more likely to have IgA titers ≥16. No significant differences in seropositivity were found, however, based on smoking behaviors.

### *Streptococcus bovis *and colorectal cancer

Colorectal cancer (CRC) is a common malignancy in developed countries and is the 3rd most common cancer in the United States [38]. Greater than 80% occur sporadically [43]. The American Cancer Society estimates that there will be about 104,950 new cases of colon cancer and 40,340 new cases of rectal cancer in 2005 in the United States. Combined, they will cause about 56,290 deaths. The risk of colon cancer increases after the age of 40 and rises exponentially from the ages of 50 to 55. In fact, more than 9 out of 10 people found to have colorectal cancer are older than 50 [38].

Survival of CRC is related to the stage of disease at the time of the initial diagnosis. Between 1985 and 1997, death rates of colon cancer in the United States declined slightly due to earlier detection of primary tumors, via stool blood tests, sigmoidoscopy, colonoscopy, and screening tests for serum carcinoembryonic antigen concentration (CEA) [44]. The 5-year survival rate for CRC patients is greater than 90% when tumors are detected at a localized early stage. After the cancer has spread regionally and involves adjacent organs or lymph nodes, the rate drops to 40–65%; survival is less than 10% for patients with distant metastases. Therefore, there is an urgent need to develop effective treatment strategies to reduce morbidity and mortality. Surgery is currently the primary treatment modality for this disease. By the time the patient presents with recurrent symptoms, however, the disease is rarely curable by surgery even when combined with other therapies [45].

Several species of bacteria have been linked to chronic infections of the colon and increased risk of colon cancer including *Escherichia coli *[46] and several streptococci [47,48]. Recent studies, however, have validated earlier findings of an association between colon cancer and *Streptococcus bovis *[11,12]. As early as 1951, McCoy and Mason [49] suggested a relationship between colonic carcinoma and the presence of infectious endocarditis. It was not until 1974 [50] that the association of *Streptococcus bovis *and colonic neoplasia was recognized, as 25–80% of patients who presented with a *S. bovis *bacteremia had a colorectal tumor. The incidence of *S. bovis *associated colon cancer has been determined as 18% to 62% [14].

*S. bovis *is a normal inhabitant of the human gastrointestinal tract that can cause bacteremia, endocarditis, and urinary infection [51]. Although *S. bovis *is the 2^nd ^greatest cause of infectious endocarditis from streptococci [50], it is frequently associated with gastrointestinal lesions, especially carcinoma of the colon [12,51-53]. Notably, the colonic neoplasia may arise years after the presentation of the condition of bacteremia or infectious endocarditis [12].

A retrospective review of forty-five documented cases of *S bovis *bacteremia was conducted by Gold et al. [12]. Subjects were identified by a search of computerized bacteriology records from one tertiary referral hospital and 1 community hospital located in the same city. Patient records were reviewed to identify the presence of colonic neoplasia, the use of gastrointestinal endoscopy, and the presence of gastrointestinal or extraintestinal malignancies. Seventeen patients (41% of adult patients) underwent colonoscopy. Colonic neoplasia was present in 16 patients (39% of adults). Invasive cancer was present in 13 patients (32% of adults), 8 of these had malignant lesions arising within the gastrointestinal tract, 3 affecting the colon and 5 patients had extraintestinal malignancies. The authors concluded that *S. bovis *bacteremia was associated with both colonic neoplasia and extracolonic malignancy.

It has been demonstrated that *S. bovis *or its wall extracted antigens (WEA) were able to promote carcinogenesis in rats [12]. In one of these investigations a total of 10 adult rats received i.p. injections of the carcinogen azoxymethane (AOM) (15 mg/kg body weight) once per week for 2 weeks. Fifteen days after the last injection of AOM (week 4) the rats were randomly divided into three groups. Twice per week during 5 weeks, the rats received, by gavage either *S. bovis *(10^10 ^bacteria Group I), WEA (100 μg Group II) and controls (Group III).

One week after the last gavage (week 10), they found that administration of either *S. bovis *or its antigens promoted the progression of preneoplastic lesions. There were increased formations of hyperproliferative aberrant colonic crypts, enhanced expression of proliferation markers and increased production of IL-8 in the colonic mucosa. Normal rats treated with the bacteria did not develop hyperplastic colonic crypts, however. The authors concluded that *S. bovis *exerts its pathological activity in the colonic mucosa only when preneoplastic lesions are established.

Under identical experimental conditions *Streptococcus gordonii *was substituted for *S. bovis*. The number of preneoplastic lesions in the colon of *S. gordonii*-treated rats was similar to rats treated with AOM alone (22 ± 2). The authors suggested that *S. bovis *and its wall extracted antigens, unlike *S. gordonii*, act as promoters of carcinogenesis in a chemically-induced animal model.

In another investigation Biarc et al. [11] isolated 12 *S. bovis *cell-associated proteins (S300) and WEA. Cells of the human colonic epithelial cell line Caco-2 originally derived from an adenocarcinoma were grown to confluence and allowed to differentiate. These cells were stimulated with 200 ul of either *S. bovis *WEA (50 μg/ml) or cell-associated proteins S300 (100 μl).

The purified S300 fraction was able to trigger the human cell line and rat colonic mucosa to release chemokines (human IL-8 or rat CINC/GRO) and prostaglandin E_2 _(PgE_2_). The 12 *S. bovis *proteins were highly effective in the promotion of pre-neoplastic lesions in azoxymethane treated rats. In fact the S300 proteins were able to induce a 5-fold increase in PGE_2 _secretion from Caco-2 cells, as compared with cells stimulated with WEA. The study found that PGE_2 _release in the human cells correlated with an over-expression of cyclooxygease-2 (COX-2).

Evidence has shown that over-expression of COX-2 has a major role in mucosal inflammation [47] and is associated with inhibition of apoptosis [54] and enhancement of angiogenesis [55], which favor cancer initiation and development. It was reported by Biarc et al. [11] that *S. bovis *proteins also promoted cell proliferation by triggering mitogen-activated protein kinases (MAPKs), which can increase the incidence of cell transformation, the rate of genetic mutations and up-regulate COX-2. The investigators concluded that colonic bacteria such as *S. bovis *can contribute to cancer development particularly in chronic infection/inflammation diseases where bacterial components may interfere with cell function [11].

## Genetic predisposition to cancer-causing infections

Research has shown that some populations are genetically predisposed to the infections that are associated with cancer and indeed have a higher risk of the cancer in question. The exact mechanisms remain unclear [38].

### *E. coli*, crohn's disease and colon cancer

Inflammatory bowel disease (IBD) includes both ulcerative colitis (UC) and Crohn's disease (CD). Both of these disorders have an increased risk of colorectal cancer (CRC) [38,46,56]. Although colorectal cancer (CRC) in individuals with IBD only accounts for 1–2% of all cases of CRC in the general population, it is considered a serious complication of the disease and accounts for approximately 15% of all deaths in patients with IBD. The magnitude of the risk has been found to differ, however, in population-based studies [56-58]. Recent figures suggest that the risk of colon cancer for people with IBD increases by 0.5–1.0% yearly, 8–10 years after diagnosis. The magnitude of CRC risk also increases with early age at IBD diagnosis, longer duration of symptoms, and extent of disease, with pancolitis having more severe inflammation and a higher risk of dysplasia-carcinoma progression [56].

*E. coli *are found at higher levels in inflammatory bowel disease (IBD), therefore, studies have examined the mechanisms that may explain this phenomenon. A cell culture study by Martin et al [46] attempted to quantify and characterize mucosa-associated and intramucosal bacteria, particularly *E. coli*, in these inflammatory conditions. Their hypothesis was that the disease-associated alterations in mucosal glycosylation found in inflammatory bowel disease and colon cancer might predispose to altered recruitment of bacteria to the mucosa.

Mucosa-associated bacteria were isolated from biopsy samples of Crohn's disease, (n = 14); ulcerative colitis, (n = 21); noninflamed controls, (n = 24) and at surgical resection of colon cancer, (n = 21). Results found that mucosa-associated and intramucosal bacteria were cultured more commonly in Crohn's disease (79%, P = 0.03; and 71%, P < 0.01, respectively), and colon cancers (71% and 57%) than in noninflamed controls (42% and 29%) but not ulcerative colitis (38% and 48%). Mucosa-associated *E. coli*, which accounted for 53% of isolates, were more common in Crohn's disease (6/14; 43%) than in noninflamed controls (4/24, 17%), and intramucosal *E. coli *more common in Crohn's disease (29%; controls, 9%).

*E. coli *expressed hemagglutinins in 39% of Crohn's cases and 38% of cancers but only 4% of controls, and this correlated (P = 0.01) with adherence to embryonic intestinal cells (I407) and colon adenocarcinoma cells (HT29). Although close apposition of *E. coli *resulted in release of pro-inflammatory cytokines, cellular invasion by bacteria was not essential to this process [46].

Aspinell [59] suggested that the bacterial adherence found by Martin et al. [46] might result from activation of virulence genes following contact of the organisms with the inflamed mucosal cells. Martin et al. [46] found, however, that the mucosal isolates expressed of none of the known virulence genes, other than adherence genes. Martin and co-workers concluded that their findings supported a central role for mucosally adherent bacteria in the pathogenesis of Crohn's disease. They postulated that similar, lower grade, inflammatory changes could contribute to the risk of sporadic cancer development [46].

The authors stated however that it was certainly possible that the presence of the bacteria in the sub-mucus niche in human Crohn's disease and colon cancer could have been encouraged by disease-associated changes [46] in the mucosa. If true, their findings would result from colonization coincidental to the disease-associated alterations in mucosal glycosylation found in inflammatory bowel disease and colon cancer.

A study conducted by Masseret et al.[60] examined the *E. coli *strains isolated from patients with Crohn's disease (CD) with chronic ileal lesions (n = 14), early endoscopic recurrent lesions (n = 20), without endoscopic recurrence (n = 7), and controls (n = 21). Genetically linked *E coli *strains were isolated significantly more frequently from patients with chronic and recurrent CD (24/33 patients) than from controls (9/21) (p < 0.05). Most patients operated on for chronic ileal lesions (78.5%) harbored *E coli *strains belonging to the same cluster (p < 0.002 v controls). The prevalence of patients with early recurrent lesions harboring *E coli *strains belonging to this cluster was high but not significant. 21 of 26 strains isolated from patients with active CD demonstrated adherent ability to differentiated Caco-2 cells, indicating that most of the genetically related strains shared a common virulence trait. Comparison of *E coli *strains recovered from ulcerated and healthy mucosa of patients operated on for CD demonstrated in each patient that a single strain colonized the intestinal mucosa. The authors suggested that although a single *E coli *isolate was not found in Crohn's ileal mucosa, some genotypes were more likely than others to be associated with chronic or early recurrent ileal lesions.

### *S. typhi *and susceptible populations

As previously stated, certain populations have an increased risk of gallbladder cancer (GC), however certain individuals may be predisposed to *S. typhi *infection which appears to increase the risk of GC. In an investigation by deJong et al. [61], three unrelated individuals with severe, idiopathic mycobacterial and *Salmonella *infections were found to lack IL-12Rβ1 chain expression. Interleukin-12 (IL-12) is a cytokine that promotes cell-mediated immunity to intracellular pathogens, such as *S. typhi*, by inducing type 1 helper T cell (T_H_1) responses and interferon-γ (IFN-γ) production. IL-12 binds to the high-affinity β_1_/β_2 _heterodimeric IL-12 receptor (IL-12R) complexes on T cell and natural killer cells. The cells of these patients were deficient in IL-12R signaling and IFN-γ production and their remaining T cell responses were independent of endogenous IL-12. IL-12Rβ_1 _sequence analysis revealed genetic mutations that resulted in premature stop codons in the extracellular domain. The genetic absence of IL12-Rβ_1 _expression represented an immune deficiency in these 3 patients. Interestingly, these patients did not develop any abnormal infections with other viral, bacterial, or fungal pathogens. The defect in IFN-production and extreme susceptibility to mycobacterial and *Salmonella *infections in these patients appeared to be a direct result of their lack of IL-12R expression and signaling. The authors concluded that selective susceptibility to mycobacterial and *Salmonella *infections, however, suggested that the type-1 cytokine pathway was essential for controlling resistance to the intracellular pathogens and that no redundant protective immune mechanism could compensate for this deficiency.

### Respiratory conditions and increased susceptibility to lung cancer

75–90% of people who develop lung cancer are smokers, however, only a small proportion of smokers develop lung cancer [42]. Hence, epidemiological studies such as that of Littman et al [42] and Kocazeybek et al. [19] have been conducted to more closely identify risk factors. Identifying genetic factors that increase a smoker's risk of developing lung cancer may help scientists to better understand the etiology of lung cancer and more effectively target high-risk groups for screening. Additionally, genetic factors have been identified that appear to predict the prognosis of certain lung cancer patients [62]. For example, mutations affecting the epidermal growth factor receptor (EGFR) were significantly associated with specific genetic alterations. Supervised clustering analysis based on *EGFR *gene mutations elucidated a subgroup including all *EGFR *gene mutated tumors, which showed significantly shorter disease-free survival

To analyze the genetic alterations of primary lung adenocarcinoma in a high-throughput way, Shibata et al. [62] used laser-capture micro-dissection of cancer cells and array comparative genomic hybridization focusing on 800 chromosomal loci containing cancer-related genes. They identified a large number of chromosomal numerical alterations, including frequent amplifications. Three subgroups of lung adenocarcinoma were characterized by distinct genetic alterations and were associated with smoking history and gender. The authors concluded that multiple carcinogenic pathways exist; certain abnormalities appear related to gender and smoking while others may impact survival [62].

## Bacterial strategies: cell cycle control and toxic warfare

Bacterial toxins can kill cells or at reduced levels alter cellular processes that control proliferation, apoptosis and differentiation. These alterations are associated with carcinogenesis and may either stimulate cellular aberrations or inhibit normal cell controls. Cell-cycle inhibitors, such as cytolethal distending toxins (CDTs) and the cycle inhibiting factor (Cif), block mitosis and are thought to compromise the immune system by inhibiting clonal expansion of lymphocytes. In contrast, cell-cycle stimulators such as the cytotoxic necrotizing factor (CNF) promote cellular proliferation and interfere with cell differentiation [20].

Bacterial toxins that subvert the host eukaryotic cell cycle have been classified as cyclomodulins. For example, CNF is a cell-cycle stimulator released by certain bacteria, such as *E. coli*. CNF triggers G_1 _– S transition and induces DNA replication. The number of cells does not increase, however. The cells become multinucleated instead, perhaps by the toxin's ability to inhibit cell differentiation and apoptosis [63,64].

Conversely the cytolethal distending toxin (CDT), as previously mentioned, is a cell-cycle inhibitor used by several species of Gram-negative bacteria, including *Campylobacter jejuni *and *S. typhi*. The CdtB unit of CDT is a DNAse that creates double-stranded DNA breaks causing cell cycle arrest, usually at the G_2 _checkpoint [65]. Cif is a cell cycle inhibitor found in enteropathogenic (EPEC) and enterohaemorrhagic (EHEC) *E. coli*. EPEC and EHEC deliver this novel toxin by injecting it into the infected epithelial cells. Cif arrests the cells at the G2/M phase [66]causing unique alterations in the host cell that result in attachment of the cytoskeleton to the host cell membrane. This anchoring of the cytoskeleton inhibits mitosis, causing cellular and nuclear enlargement. Although DNA synthesis is initiated it does not lead to nuclear division. Endoreduplicaton occurs resulting in cellular DNA content of 8–16n [20,66].

In a cell culture study, Haghjoo and Galán [65] found that *S. typhi *produced a unique *cdtB*-dependent CDT that required bacterial internalization into host cells. When Cos-2 cells were transfected with *S. typhi *the effects of the *cdtB *subunit were severe fragmentation of chromatin characteristic of the CdtB subunit of CDT expressed by other species. The authors proposed that *S. typhi *subsequent to internalization deviated from the usual endocytic pathway that leads to lysosomes, reaching an unusual membrane-bound compartment where it can survive and replicate. It is possible that this unique CDT may be involved in some aspects of the ability of *S. typhi *to cause long, persistent infections in humans, because, at least in other bacteria, this toxin has been shown to possess immunomodulatory activities.

Toxins are not the only strategy for evading the host's immune system, however. An early study by Kilian et al. [67] reported that some strains of *Capnocytophaga ochracea*, an oral pathogen, are capable of hydrolytically degrading immunoglobulin A subclass 1 found in the oral cavity. This property may enhance colonization and invasion of oral lesions which characterize many bacteremias due to *Capnocytophaga *species. [67]. Shurin et al. [68] obtained evidence that Capnocytophaga species inhibit polymorphonuclear leukocyte migration; a means by which these species may evade phagocytosis.

The immune system may also be evaded by the protection offered by bacterial biofilms. An example of this phenomenon is provided by uropathic *E. coli *species whose biofilms protect it from the immune system and making it difficult to treat these infections effectively by antibiotics. This has been demonstrated in bladder infections where the same species is recovered after repeated flare-ups thought to have been cleared by antibiotic therapy, suggesting a subclinical infection that has become chronic [69].

## Bacterial site-specific colonization

Bacterial adherence is thought to be the first important step in colonization. It is now recognized that bacteria bind to and colonize host cells in a highly selective manner via a "lock- and key" mechanism. This selectivity of bacterial adhesion plays an important role in many infectious processes, and an understanding of the mechanisms involved could provide molecular explanations for the innate resistance or susceptibility of hosts and tissues to many infectious agents.

Regulators of complement activation (RCA proteins) prevent the destructive consequences of inappropriate immune activation. Decay-accelerating factor (CD55) is a member of the RCA protein family that protects host cells from complement damage and regulates the classical, alternative and lectin pathways that converge to target cells for destruction in all 3 pathways of the innate immune system [70]. CD55 is expressed on all serum-exposed cells. Perhaps due to its ubiquitous expression, it is thought that bacterial pathogens, including uropathogenic *Escherichia coli*, use CD55 as a receptor prior to infection. Williams et al. [70] suggested that pathogens have evolved to exploit the cellular roles of this molecule thereby gaining immunological advantage [70].

The influence on *E. coli *binding of the two known single amino acid polymorphisms within short consensus repeat (SCR) domains of CD55 was examined by Pham et al. [71] and Nowicki et al [72]. The bacterial strains sensitive to a change in SCR3 were found to be insensitive to changes in SCR4 and vice versa, suggesting that multiple, independent binding sites of CD55 were used by different bacterial strains. Evidence from those investigations suggested that *E. coli *strains sensitive to changes in one binding domain were not affected by changes in other domains.  Furthermore, the use of CD55 as a receptor by a variety of uropathic *E. coli *was found to correlate with symptomatic infections [71,72]. Evidence from those investigations indicated the extraordinary degree of site-specific colonization of these closely related strains.

## Bacteria associated with a coincidental or diagnostic role

Each year nearly 30,000 Americans are diagnosed with oral cancer [73,74]. Over 90% of these malignancies are oral squamous cell carcinoma (OSCC). Despite advances in surgery, radiation and chemotherapy, the five-year survival rate is 54%, one of the lowest of the major cancer sites and this rate has not improved significantly in recent decades [38,75,76]. The disease kills one person every hour – more people than cervical cancer, Hodgkin's disease, or malignant melanoma [38]. Notably, incidence in young adults (<40 years) is increasing in the U.S. [8,10] and worldwide [9,77]. The World Health Organization predicts a continuing worldwide increase in oral cancer over the next several decades [78].

Early detection followed by appropriate treatment, increases cure rates to about 80%, and greatly improves the quality of life by minimizing extensive, debilitating treatments [75]. Oral cancer is asymptomatic in its early stages, however, and in spite of the accessibility of the oral cavity to direct examination, these malignancies are often not detected until a late stage [79-81]. Oral cancer is unusual in that it carries a high risk of second primary tumors. Patients who survive a first cancer of the oral cavity have up to a 20-fold increased risk of developing a second primary oral cancer. The heightened risk can last 5–10 years, sometimes longer [82].

In response to the difficulties in effectively treating oral cancer, research studies are focusing on prevention and early diagnostics. Some of these studies have found that OSCC lesions are colonized by an altered microbiota [83,84]. Other investigations have found bacterial DNA or live organisms within oral cancer tissues [85,86]. The true nature of the relationships between oral bacteria and oral or esophageal cancers is, however, currently unknown.

PCR techniques have been used to seek the DNA of bacterial species in head and neck cancer tissues. Sasaki et al. [85] found *S. anginosus *DNA sequences in tissue samples from 127 cancer patients. Tissues examined included esophageal cancer, gastric cancer tissues, and dysplasia of the esophagus from esophageal cancer patients. No *S. anginosus *DNA was found in noncancerous esophagus or stomach samples. However, the degree of *S. anginosus *infection in biopsied tissues was much more obvious in the dysplastic and cancerous sections than in the noncancerous portions of the esophagus suggesting that *S. anginosus *infection occurred at an early stage of esophageal cancer. The authors suggested that *S. anginosus *could play a significant role in the carcinogenic process of most cases of esophageal cancer and some cases of gastric cancer by causing inflammation.

Morita et al. [86] found that 8 of 18 (44%) samples from the esophagus contained a detectable level of *S. anginosus *DNA, but only 5 of 38 (13%) of oral cancer had detectable DNA levels of this organism. The quantity of *S. anginosus *DNA in the esophageal cancer tissues was significantly higher than in oral cancer. The maximum amount of *S. anginosus *DNA was approximately 10 times higher in esophageal than in oral cancer tissues. In addition, none of the 5 different oral cancer sites (floor of mouth, maxillary or mandibular gingiva, buccal mucosa, and tongue) showed significant signs of *S. anginosus *infection. Most non-cancerous tissues of the esophagus and tongue showed an undetectable level of *S. anginosus*. The authors concluded that *S. anginosus *is associated with esophageal cancer, but is not closely related with oral cancer.

In a previous study by Mager et al. [87] it was determined that the salivary microbiota was similar to that of the oral soft tissues. Therefore, the investigators examined whether the salivary counts of 40 common oral bacteria in subjects with an oral squamous cell carcinoma (OSCC) lesion would differ from those found in cancer-free (OSCC-free) controls [83]. Unstimulated saliva samples were collected from 229 OSCC-free and 45 OSCC subjects and evaluated for their content of 40 common oral bacteria using checkerboard DNA-DNA hybridization.

DNA counts per ml saliva were determined for each species, averaged across subjects in the 2 subject groups, and the significance of differences between groups determined using the Mann-Whitney test and adjusted for multiple comparisons. The diagnostic sensitivity and specificity in detection of OSCC by levels of salivary organisms were computed and comparisons made separately between a non-matched group of 45 OSCC subjects and 229 controls and a group of 45 OSCC subjects and 45 controls matched by age, gender and smoking history.

Counts of 3 of the 40 species tested, *Capnocytophaga gingivalis*, *Prevotella melaninogenica *and *Streptococcus mitis*, were elevated in the saliva of individuals with OSCC (p < 0.001). When tested as diagnostic markers the 3 species were found to predict 80% of cancer cases (sensitivity) while excluding 83% of controls (specificity) in the non-matched group. Diagnostic sensitivity and specificity in the matched group were 80% and 82% respectively. These findings suggest that high salivary counts of *C. gingivalis*, *P. melaninogenica *and *S. mitis *could be diagnostic indicators of OSCC.

The reasons for the differences in colonization patterns of specific bacterial species at different host locations are only partially understood. These reasons include differences in nutrient availability, competition among species for binding sites, inter-species antagonisms or cooperations, and the differences in receptors present on different tissues that permit binding by specific adhesins possessed by different species. Other factors that may partly explain the unfavorable microbial shifts observed in oral carcinoma surface biofilms are a compromised host response or the irregularity of the lesion surface providing stagnant habitats.

The most intensely studied of these possibilities has been the specificity in adhesion of different bacterial species to receptors on oral soft tissues. Many studies have focused on fimbriae-mediated adhesion and adhesins in the adherence of different oral species to oral epithelial cells [88-91]. As a universal trait of cancer cells is alterations in cell surface receptors, studies have examined the colonization of healthy and cancerous epithelia [83,85-87,92].

A study by Neeser et al. [92] examined the binding of a common oral bacterial species, *Streptococcus sanguis *OMZ 9 to healthy and cancerous buccal cell lines. Results showed that *S. sanguis *bound to exfoliated human buccal epithelial cells in a sialic acid-sensitive manner. The desialylation of such cells invariably abolished adhesion of *S. sanguis *to the epithelial cell surface. The resialylation of desialylated HBEC with CMP-sialic acid and Galß1,3GalNAc α2,3-sialyltransferase specific for *O*-glycans restores the receptor function for *S. sanguis *OMZ 9, whereas a similar cell resialylation with the Galß1,4GlcNAc α2,6-sialyltmnsferase specific for *N*-glycans is without effect. These findings suggested that a 23 kDa cell surface glycoprotein bearing a carbohydrate sequence, NeuNAc alpha 2-3Gal beta 1-3GalNAc O-linked sugar chains, is recognized by *S. sanguis *on exfoliated human buccal epithelial cells. In similar experiments carried out with a buccal carcinoma cell line termed SqCC/Y1, *S. sanguis *did not attach in great numbers to cultured tumor cells. These cells were shown to not express the membrane glycoprotein bearing alpha 2,3-sialylated O-linked carbohydrate chains.

Aberrations in the cell surface carbohydrate structures have now been established as a universal characteristic of malignant transformation of cells, and cancer has been referred to as a molecular disease of the cell membrane glycoconjugates [93,94]. Thus, changes in the tumor cell surface structure could alter the adhesion of different species of oral bacteria. Notably, even species within the same genera, such as streptococci, have been found to differ in their colonization of healthy and cancerous oral tissues [83,87].

## Bacteria and the prevention or treatment of cancer

Evidence is mounting that certain species of bacteria or their toxins may indeed have a protective or curative role in some cancers. Factors that would suggest a protective role of a bacterial species include: (1) colonization lowers the risk of a certain cancer; (2) elimination or absence of colonization raises the risk, or (3) introduction of the bacteria or its toxins cures or causes remission of the cancer.

### Tumors and coley's toxins

Spontaneous tumor regression has followed severe bacterial, fungal, viral and protozoal infections. For hundreds of years this phenomenon inspired the development of the earliest cancer therapies. Reports of spontaneous remissions of advanced cancers infections can be found in the late nineteenth and early twentieth centuries. Many of these unexplained cures followed bacterial infections accompanied by high fevers.

An American surgeon, Dr. William Coley began the first well-documented use of bacteria and their toxins to treat end stage cancers. Coley first used live *Streptococcus pyogenes *cultures. Problems with the predictability of patient responses caused him to develop a safer vaccine in the late 1800's composed of two killed bacterial species, *S. pyogenes *and *Serratia marcescens*. In this way he could simulate an infection with the accompanying fever without the risk of an actual infection [95,96].

Coley's vaccine was widely used to successfully treat sarcomas, carcinomas, lymphomas, melanomas and myelomas. Complete, prolonged regression of advanced malignancy was documented in many cases. The combined reports of Coley and others estimated the 5-year survival rate at 80% in malignancies for which no treatment existed. Even in patients considered in the terminal stages of cancer some remarkable recoveries were reported with the patient often outliving the cancer [97].

Coley considered 4 points critical to success: (1) initiation of a naturally occurring infection with fever, (2) avoidance of immune tolerance by gradually increasing the dosage, (3) directly injecting the vaccine into the tumor when accessible, and (4) a minimum of 6 months of injections to avoid recurrences. Today little credence is given to the febrile response in fighting cancer [96,97].

A retrospective study was conducted in 1999 to compare the 10 year survival rate of patients treated by Coley's vaccine with modern conventional therapies. Richardson et al. [95] tried to match 128 of Coley's cases with 1,675 controls from the Surveillance Epidemiology End Result (SEER) cancer registry. The 2 populations were matched by age, gender, ethnicity, stage and radiation treatment status. Limitations included sample size and staging of patients receiving Coley's vaccine. The authors concluded that "Given the tremendous advances in surgical techniques and medicine in general, any cohort of modern patients should be expected to fare better than patients treated 50 or more years ago. Yet no such statistical advantage for the modern group was observed in this study." These findings were supported by case reports of spontaneous remissions or significant benefits when accidental infections occurred [98-100].

What role may a febrile response play in the remission of a tumor? Hobohm [101] offers the following hypothesis. Fever causes a cascade of events of inflammatory factors which activate resting dendritic cells (DC) that lead to the activation of T-cells. Cancer-cell specific T-cells usually remain in a state of anergy, most likely due to the absence of danger signals that usually accompany tissue destruction and inflammation upon acute infection [102]. A feverish bacterial infection may have a 3-fold beneficial effect. First, many infectious agents release endotoxins, like LPS, induce inflammatory cytokines and stimulate DC. Second, both thymocyte proliferation and generation of allo-specific CTL are increased with temperature in vitro [103]. Third, the vasculature of a tumor is more fragile than that of normal tissues and therefore more prone to destruction by the immune response. An infection causing hemorrhagic necrosis could trigger febrile collapse of the tumor vasculature [104,105]. Interestingly, the affinity of certain streptococci for binding to fibrinogen and fibrin may account for the 'homing' of bacterial enzymes to tumors as these cells are abundant in such proteins [106].

The mechanism by which infection cures cancer has been investigated. It has been suggested by Zacharski and Sukhatme [96] that the tumor regression observed by Coley and others is due to the activation of plasminogen. For example when the streptococcal spreading factor known as streptokinase (SK) combines with host plasminogen, plasmin is released. Plasmin triggers protease cascades that degrade plasma and extracellular matrix proteins. These mechanisms of degradation are toxic to tumor cells, disrupt the tumor extracellular matrix, alter tumor growth and inhibit metastasis [96]. The notion that plasminogen activators like SK might result in the remissions reported by Coley is appealing as they appear to spare healthy cells while attacking tumors. Zacharski et al. [107] hypothesized that although the potent enzymes produced by plasminogen activation may have a direct effect on cancer cells it was more likely they disrupted the cell-extracellular matrix of the tumor.

Investigators report that the traditional best treatment options for some candidate tumor types, such as advanced soft tissue sarcomas, breast cancer and melanoma, have not improved patient outcome substantially since Coley's day [96,108,109]. Currently, biologic response modifier therapies have moved beyond the nonspecific immunotherapy of Coley's era and laid the foundation for today's approaches. Zarcharski and Sukhatme [96] suggest that the early success of Coley's toxins are leading to therapies that engage the host's immune system against an individual's tumor offering new hope for cancer patients.

Autologous tumor cell vaccine therapy is an example of this new approach to cancer treatment. These vaccines differ markedly from conventional cytotoxic drug therapy that affect both normal and tumor cells. Tumor vaccines stimulate an individual's cell-mediated immune response by targeting the patient's tumor antigens. While efficacy of standard chemotherapy relates to the dose of the drug, the efficacy of a tumor vaccine is more complex, involving host-vaccine interactions [110]. These include: (1) immunogenicity of the vaccine regarding tumor-associated antigens as opposed to self; (2) the host's immune response in terms of immune recognition and effector mechanisms; and (3) the development of host systemic cell-mediated immunity, including long-term immunologic memory, (3–5 years). Therefore, the potency of the vaccine is not determined by immunogenicity alone but by its ability to induce the host anti-tumor response [110].

### Bacillus calmette-guérin and autologous tumor cell vaccine vs. colon cancer

Certain tumor antigens are, however, normally weak immunogens. Therefore the use of adjuvants and the intradermal route of injection have, in some cases, produced an optimum antigenic vaccine. These adjuvant vaccines have induced effective host recognition of tumor-associated antigens and improved patient survival. For example, preliminary evidence by Hoover et al [111] suggested that active specific immunotherapy (ASI) of colon cancer using autologous tumor cell vaccines had potential in improving recurrence-free interval and survival. ASI assumes there are distinct tumor antigens on an individual's cancer cells that are either absent or in lower concentration on normal cells. The vaccine attempted to stimulate host's immune defenses against tumor-associated antigens by enhancing the immunogenicity of the patient's own tumor cells with an immunomodulating adjuvant, such as *Bacillus Calmette-Guérin *(BCG).

In a study by Hoover et al. [111] 80 eligible subjects with colon (47) or rectal (33) cancer were enrolled into a prospectively randomized, controlled clinical trial of active specific immunotherapy (ASI). An autologous tumor cell-Bacillus Calmette- Guerin (BCG) vaccine was used to determine whether ASI could improve disease- free status and survival. Eligible subjects had colon or rectal cancers extending through the bowel wall or had positive lymph nodes providing adequate cells from the primary tumor. Wide surgical removal of all tumors was performed with histologically proven clear margins and removal of involved lymph nodes. Prior to randomization individuals were screened for metastatic disease. Colon cancer and rectal cancer subjects were in separate but parallel studies and randomized into groups treated by resection alone or resection plus ASI. 3–4 weeks following surgery, both controls and treatment subjects were skin tested for immune competence and sensitivity to tuberculin purified protein derivative (PPD). Vaccines were begun in the ASI treatment group 4–5 weeks following surgery to allow for adequate immune recovery from surgery and anesthesia. A total of 24 colon and 17 rectal subjects composed the treatment group. With a median follow-up of 93 months, there was a significant improvement in survival (two-sided P = .02; hazards ratio, 3.97) and disease-free survival (two-sided P = .039; hazards ratio, 2.67) in all eligible colon cancer patients who received ASI. With a median follow-up of 58 months, no benefits were seen in patients with rectal cancer who received ASI. The authors concluded that the study suggested that ASI may be beneficial to patients with colon cancer.

In 2005, Uyl-de Groot et al. [110] conducted a multicenter, randomized controlled phase III clinical trial with Stage II and III colon cancer patients using ASI. Autologous tumor cells were used with the immunomodulating adjuvant *Bacillus Calmette-Guérin *(BCG) in a vaccine (OncoVAX^®^). Patients were randomized to receive either OncoVAX^® ^or no therapy after surgical resection of the primary tumor. The vaccine was processed within 48 h after surgery in order to have viable, metabolically active, autologous tumor cells.

Analysis of prognostic benefit with a 5.8 year median follow-up, showed that the beneficial effects of OncoVAX^® ^were statistically significant at all endpoints including recurrence-free interval, overall survival, and recurrence-free survival in Stage II colon cancer patients. Surgery alone cures 65% of Stage II colon cancer patients. For the remaining patients, OncoVAX^® ^in an adjuvant setting significantly prolongs recurrence-free interval and significantly improves 5-year overall survival and recurrence-free survival. Unfortunately, no statistically significant prognostic benefits were achieved in Stage III patients [110].

### Immunization with bacillus calmette-guérin vs. lung cancer

Grant et al [39] hypothesized that optimal chemotherapy with or without radiation followed by active immunization could eliminate microscopic residual disease and prolong survival. Immunization with GD3, a ganglioside expressed on the surface of most small cell lung cancers (SCLC) had not evoked a strong immune response. Therefore BEC2, a large xenogenic protein which mimics GD3, was judged to be a good immunogenic candidate. This approach had proven successful in extending the lives of melanoma patients [112].

Chapman et al. [113] conducted a phase II trial comparing 5 dose levels of BEC2. The study population consisted of 15 patients with small cell lung cancer (SCLC). All subjects had completed standard therapy and had achieved a partial or complete response. Patients received a series of five intradermal immunizations consisting of 2.5 mg of BEC2 plus BCG over a 10-week period. Blood was collected for serological analysis, and outcome was monitored. All patients developed anti-BEC2 antibodies, despite having received chemotherapy with or without thoracic radiation. Anti-GD3 antibodies were detected in five patients, including those with the longest relapse-free survival. The median relapse-free survival for patients with extensive stage disease was 10.6 months. In patients with limited stage disease a median relapse-free survival had not been reached with a follow-up of >47 months and only one of the 7 patients in this group relapsed. The authors reported that immunization of SCLC patients using BEC2 plus BCG after standard therapy could induce anti-GD3 antibodies and was safe. The survival and relapse-free survival in this group of patients was substantially better than those observed in similar patients receiving standard therapy.

A Phase III trial was conducted to evaluate BEC2 plus BCG as adjuvant therapy for limited small-cell carcinoma after chemotherapy and irradiation [114]. A total of 515 subjects were randomly assigned. Unfortunately, in this trial there was no improvement in survival, progression-free survival, or quality of life in subjects that were vaccinated. A trend toward prolonged survival was observed in the one third of subjects who developed a humoral response (p = 0.085), however.

The effectiveness of vaccines for several cancers was examined in a series of investigations. Xiang et al. [115] tested vaccines for human melanoma using the mutant *S. typhi *strain SL7207 as a DNA carrier. Tolerance against self-antigens was broken by genetically fusing ubiquitin with MHC I derivatives. Another approach coupled tumor-specific antibodies to functional IL-2. This combination in addition to oral vaccination with plasmid-encoded tumor antigens significantly enhanced protection against carcinoma of the colon [116], carcinoma of the lung [117] and melanoma [118]. Unstable cancer cells provided a challenge, however. Interestingly, this was overcome by targeting stable, proliferating endothelial cells of the tumor vasculature. This novel approach effectively inhibited angiogenesis [119].

### *Helicobacter pylori *and esophageal adenocarcinoma

In industrialized countries the incidence of *H pylori *has been steadily decreasing [120]. The incidence of esophageal cancer (EA), however, is increasing [121]. Surprisingly, there is evidence that these two trends may be related. Several studies have determined that virulent strains of *H pylori *are found less commonly among patients with Barrett's esophagus and EA when compared with controls [122-124]. This led to studies that found positive associations among the increased incidence of obesity, GORD, Barrett's esophagus and EA [125].

Recently, a nested case-control study was conducted by de Martel et al [126] to assess the association between *H. pylori *infection and the risk of development of EA. Of a total of 128,992 members of an integrated health care system who had participated in a multiphasic health checkup (MHC) during 1964–1969, 52 patients developed EA during follow-up. Three randomly chosen control subjects from the MHC cohort were matched to each cancer subject, on the basis of age, gender, race, date and site of the MHC. Data on cigarette smoking, alcohol consumption, body mass index (BMI), and education level were obtained. Serum samples collected at the MHC were tested for IgG antibodies to *H. pylori *and to the *H. pylori *CagA antigen associated with *H. pylori *virulence.

Subjects with *H. pylori *infections were less likely than uninfected subjects to develop EA odds ratio (OR, 0.37) 95% confidence interval (CI, 0.16–0.88). This significant association was restricted to cancer subjects and control subjects <50 years old (OR, 0.20) (95% CI, 0.06–0.68). Interestingly, in patients with *H. pylori *infections, the OR for EA in those who tested positive for IgG antibodies to the CagA protein was similar to that for those who tested negative for it. BMI ≥25 and cigarette smoking, however, were strong independent risk factors for EA. The authors found, however, that the absence of *H. pylori *infection, independent of cigarette smoking and BMI, was associated with an increase in the risk of development of EA [126].

An epidemiological study in Sweden sought to determine whether BMI was associated with esophageal malignancies compared to gastric adenocarcinoma and controls. In a nationwide, population-based case-control study by Lagergren et al [127], between 1995 through 1997, a total of 189 patients with adenocarcinoma of the esophagus and 262 patients with adenocarcinoma of the gastric cardia were enrolled. These patients were compared with 167 patients with incident esophageal squamous cell carcinoma and 820 healthy controls.

Odds ratios were determined from BMI and cancer case-control status and ratios estimated the relative risk for the two adenocarcinomas studied. Calculations used multivariate logistic regression with adjustment for potential confounding factors. The adjusted odds ratio was 7.6 (95% CI, 3.8 to 15.2) among persons in the highest BMI quartile compared with persons in the lowest. Obese persons (persons with a BMI>30 kg/m^2^) had an odds ratio of 16.2 (CI, 6.3 to 41.4) compared with the leanest persons (persons with a BMI<22 kg/m^2^). The odds ratio for patients with cardia adenocarcinoma was 2.3 (CI, 1.5 to 3.6) in those in the highest BMI quartile compared with those in the lowest BMI quartile and 4.3 (CI, 2.1 to 8.7) among obese persons. Although a strong dose-dependent relation existed between BMI and esophageal adenocarcinoma, esophageal squamous-cell carcinoma was not associated with BMI. A modest but significant increase in intragastric acidity was also observed following the cure of *H pylori *infection which the authors postulated could contribute to gastroesophageal reflux disease (GORD).

The incidence of EA has increased rapidly over the last 30 years. During this period, the prevalence of *Helicobacter pylori *has decreased. Trends of increasing esophageal adenocarcinoma can be linked causally to increasing GORD which can be linked to an increasingly obese population. There appeared to be no plausible biological mechanism of association between *H pylori*, obesity, and GORD until studies of ghrelin, however.

Ghrelin was the first circulating hormone demonstrated to stimulate food intake in man. This peptide is produced in the stomach and regulates appetite, food intake, and body composition. The effects of ghrelin were examined in *H pylori *positive asymptomatic subjects by several investigators [128-130]. In a randomized double-blind cross-over study, by Wren et al. [129], ghrelin was shown to acutely enhance appetite and increase food intake in 9 healthy human subjects. There was a clear-cut increase in calories consumed by every individual from a free-choice buffet (mean increase 28 +/- 3.9%, p < 0.001) during ghrelin versus saline infusions. Furthermore, visual analogue scores for appetite were greater during ghrelin compared to saline infusion. Ghrelin had no effect on gastric emptying, however. The authors concluded that endogenous ghrelin was a potentially important new regulator of the complex systems controlling food intake and body weight.

Evidence is accumulating that ghrelin may explain the relative rarity of *H. pylori *among patients with Barrett's esophagus and EA. Findings from these studies and others support the notion that *H. pylori *may have a "protective" effect against EA [122,124]. Studies have found that curing *H pylori *infection increased plasma ghrelin in healthy asymptomatic subjects which may lead to increased appetite, weight gain and contribute to the increasing obesity seen in Western populations where the prevalence of *H pylori *is low. This evidence supports the notion that decreasing incidence of *H pylori *infection may lead to increased levels of plasma ghrelin and that this hormone appears to be a factor in increasing obesity which elevates the risk of GORD which is positively associated with Barrett's esophagus and increased risk of esophageal adenocarcinoma. It appears that the absence of *H. pylori *infection may be one of several factors that leads to the increased incidence in EA effect observed in Western populations.

The implications for treatment of individuals with *H. pylori *infection were addressed by Nakajima and Hattori [131]. They systematically reviewed the literature and estimated the expected annual incidence of esophageal or gastric cancer with and without eradication of *H. pylori *infection in patients with chronic atrophic gastritis. The expected annual incidence of gastric cancer in patients with corpus atrophy with persistent infection was at least 5.8-fold higher than that for esophageal adenocarcinoma after the eradication of infection at all ages. Even for patients with accompanying reflux esophagitis or Barrett's esophagus, the incidence of gastric adenocarcinoma with persistent infection was higher than that of esophageal adenocarcinoma after eradication of infection. The authors concluded, therefore, that if eradication of infection lowers the incidence of gastric cancer, it should be recommended for patients with corpus atrophy at all ages irrespective of the presence of reflux esophagitis or Barrett's esophagus, especially in populations having a high prevalence of gastric cancer [131].

In summary, increased BMI has been linked with the elimination of *H. pylori *infection. As the sphincter mechanism at the esophagogastric junction is weakened by weight it is not surprising that obese individuals have a higher incidence of gastric reflux or GORD [127]. GORD may lead to the development of Barrett's esophagus, which increases the risk of EA by 40-fold [132,133]. The study by Lagergren [127] provides evidence that these associations may be related as increasing body mass was associated with a stepwise increase in the risk of EA. If eradication of *H. pylori *infection lowers the incidence of gastric cancer, however, it should be recommended for patients with corpus atrophy at all ages irrespective of the presence of reflux esophagitis or Barrett's esophagus, especially in populations having a high prevalence of gastric cancer [131].

### Attenuated bacteria: Promising carriers of DNA vaccines

Attenuated bacteria will enhance stimulation of the innate immune system yet increase the safety of a vaccine, [134] therefore they may be ideal for the delivery of vaccines. Animal studies have shown that attenuated *S. typhimurium *strains can successfully deliver a variety of genetically engineered DNA vaccine plasmids for therapeutic vaccination of mice against model tumors [117,118,135].

The identification of bacterial "carriers" for DNA vaccines that target cancer cells by site-specific colonization may allow the selective delivery of vaccine plasmids into tumor cells [136]. Colonization of these species may be considered coincidental to favorable conditions provided by the tumor yet prove clinically useful. Ultimately, however, the safety and efficacy of recombinant therapeutic agents expressed by plasmids must be conducted in appropriate animal models.

## Conclusion

Cancer is commonly defined as the uncontrolled growth of abnormal cells that have accumulated enough DNA damage to be freed from the normal restraints of the cell cycle. Several pathogenic bacteria, particularly those that can establish a persistent, infection, can promote or initiate abnormal cell growth by evading the immune system or suppressing apoptosis [54,137]. Intracellular pathogens survive by evading the ability of the host to identify them as foreign. Other species or their toxins can alter host cell cycles or stimulate the production of inflammatory substances linked to DNA damage [120].

The highly site-specific adherence of bacteria involves binding species-specific adhesions to the required cell surface receptors. The role of species that colonize tumors could be causal, coincidental or potentially protective. If adhesion to the tumor in question is highly sensitive and specific it may be ideal not only in diagnosing the presence of a malignancy but also in delivering the appropriate therapy.

The bacterial species associated with cancer etiology are diverse; however, the infections they cause share common characteristics [18]. The time between acquiring the infection and cancer development is most often years or even decades as seen in cancers associated with *H. pylori*, *S. typhi *and *S. bovis *infections. Chronic interactions between the infective agent and immune response and/or a susceptible host appear to contribute to carcinogenesis [8,18,38,138]. Preventing or treating the infection may prevent the cancer in question. Notably, the vast majority of individuals infected with a cancer-causing species will not develop cancer [18].

Evidence suggests that certain individuals are more susceptible to infections linked to cancer development and that the incidence of certain cancers varies among populations. For example, gallbladder cancer is 3 times higher in females as in males in all populations [26]. Lung cancer is highest in populations that smoke however, only a small proportion of smokers develop lung cancer [42]. Although colon cancer is the 3^rd ^highest cancer in the United States, individuals with IBD have a far greater risk of colorectal cancer than individuals without IBD [56-58].

A screening test for oral cancer based on salivary counts of bacterial species is appealing. Currently saliva is meeting the demand for inexpensive, non-invasive, and easy-to-use diagnostic aids for oral and systemic diseases, and for assessing risk behaviors such as tobacco and alcohol use. Although the colonization of certain bacterial species may be coincidental to favorable conditions provided by OSCC, increased numbers of certain salivary species may be clinically useful if shown to be a signature of oral cancer and if sensitivity and specificity are improved.

Successful treatment for cancers was reported by Dr. Coley and others one hundred years ago. His approach of using killed bacterial vaccines was surprisingly effective in some patients even in the latest stages of cancer. Dr. Coley believed that the human immune system had the power to cure cancers if properly stimulated. Today, some investigators agree and have designed new treatments that stimulate the immune system to recognize and target the lesion. Recent reports suggest that attenuated bacterial vaccines can safely and effectively deliver plasmids encoding tumor self antigens. These studies have reported successful treatment of certain cancers and prevention of recurrences [39,110,111]. Cancer vaccines although promising, present significant challenges. These include identification of highly effective bacterial strains and their attenuations, addressing safety issues and the problem of overcoming the peripheral T cell tolerance against tumor self-antigens [139]. Further, the response to vaccines will likely vary among individuals.

It appears that colonization by certain bacteria may reduce the risk of cancer in some populations. The epidemiological trends of esophageal adenocarcinoma and *Helicobacter pylori *infection have stimulated research into whether these may be coincidental or due to an inverse association. Intriguing results suggest there is an association represented by a complex continuum that begins with curing infections of virulent strains of *H. pylori*. The absence of *H. pylori *appears to elevate ghrelin which stimulates increased appetite in some individuals. High ghrelin levels appear to be associated with increased incidence of obesity.  Obesity is reported to be a contributing factor in GORD.  Finally, GORD may lead to Barrett's esophagus which increases the risk of esophageal adenocarcinoma. If these relationships can be proven, then the colonization of this species and its seemingly negative association with EA may be more clearly understood.

In summary, recent research has uncovered a great deal of information regarding the bacterial mechanisms used to cause, colonize or cure cancer, however, many questions remain. For example, do the bacteria in question initiate, promote, or merely show affinity for the neoplasm? Conversely does cancer weaken the host which facilitates acquiring the infection? Can the highly site specific colonization of certain bacteria for a tumor be clinically useful in diagnosis or treatment? Could attenuated bacteria be used in vaccines to safely and effectively deliver therapeutic agents? The continued exploration of these questions will bring research ever closer to the prevention, early diagnosis and truly effective treatment of this scourge of mankind.

## Competing interests

The author(s) declare that they have no competing interests.
